# Familial urothelial cell carcinoma of the bladder with autosomal dominant inheritance and late onset phenotype

**DOI:** 10.1186/2193-1801-3-281

**Published:** 2014-06-03

**Authors:** Robin Brown, Deirdre E Donnelly, Derek Allen, Maurice B Loughrey, Patrick J Morrison

**Affiliations:** Department of Urological Surgery, Daisy Hill Hospital, 5 Hospital Road, Newry, BT35 8DR UK; Department of Histopathology, Belfast HSC Trust, Lisburn Road, Belfast, BT9 7AB UK; Department Genetic Medicine, Belfast HSC Trust, Lisburn Road, Belfast, BT9 7AB UK; Centre for Cancer Research and Cell Biology, Queens University of Belfast, 97 Lisburn Road, Belfast, BT9 7AE UK

**Keywords:** Urothelial cell bladder, Cancer, Autosomal dominant

## Abstract

**Objective:**

Familial Urothelial cell bladder cancer is rare. We report two families with urothelial cell carcinoma (UCC) of bladder with family history in other relatives, displaying probable autosomal dominant inheritance and a late onset pure UCC phenotype, and document the phenotype in each family.

**Methods:**

Descriptive familial study on two pedigrees over three generations.

**Results:**

Two families with UCC bladder were identified, and the phenotype documented, each family having three cases of late onset UCC.

**Conclusion:**

Some cases of UCC are hereditary and may display autosomal dominant inheritance with late onset of the cancer. Clinicians should be aware of the existence of a familial late onset UCC phenotype when managing cases of UCC.

## Introduction

Urothelial cell carcinoma (UCC) of bladder is common. The main aetiological factors identified are cigarette smoking and certain occupational exposures. Familial UCC bladder is rare and infrequently encountered. Documented cases of familial UCC bladder in the medical literature are rare (Fraumeni and Thomas 1967; McCullough et al. 1975; Ilic et al. 2011) and display early onset. Some families with hereditary non-polyposis colon cancer (HNPCC) in particular due to MSH2 mutations, can also include cases of UCC bladder (van der Post et al. 2010), although these are predominantly upper tract UCC, but often cases reported in the literature have not been checked for HNPCC. We present two families with pure UCC bladder and a late onset phenotype suggesting that some types of UCC bladder may be late onset and autosomal dominant in nature.

## Subjects and methods

**Figure 1:**
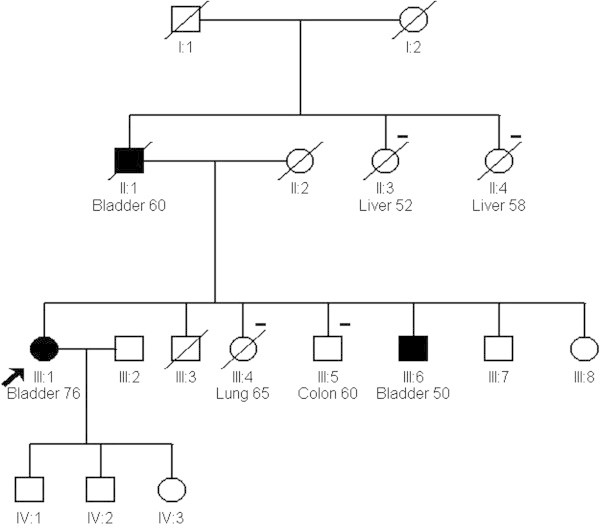
**Three generation pedigree of family A.** Bladder cancer shown as shaded. - indicates other cancer.

**Figure 2:**
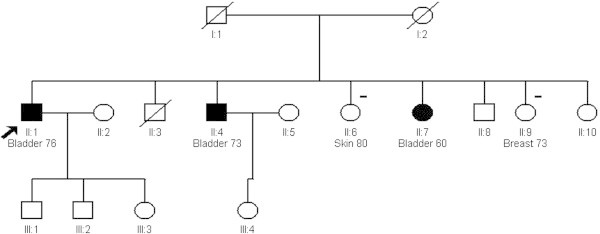
**Four generation pedigree of family B.** Bladder cancer shown as shaded. - indicates other cancer.

Detailed analysis of two families with three or more cases of urothelial cell cancer of the bladder was carried out. Neither family had any significant relevant occupational or environmental exposure. Smoking history was negative except where listed.Family A. Three cases of UCC in the same sibship presented. All were smokers in youth only. The male proband II.1 was diagnosed with WHO stage III UCC at 76 years old, his brother II.4 with stage II UCC at 73 years and his sister II.7 with stage I UCC at 60 years. A sister II.9 had breast cancer at 73 years and a sister II.6 had a basal cell skin cancer at 80 years. Their father I.1 died of heart disease at 61, and mother I.2 of ‘old age’ at 86. No parental siblings had a history of any relevant cancer (Figure 1).Family B. A male proband II.1 was diagnosed with UCC bladder at 60 years. Staging is not recorded. Two siblings II3 and II.4, developed liver cancer due to complications of hereditary ferritinaemia (HFE). The proband’s daughter III.1 developed stage III bladder cancer aged 76, a son III.6 was diagnosed with stage II UCC bladder at age 50 (also a HFE gene carrier) a daughter III.4 (a smoker) developed lung cancer at age 65, and a son III.5 developed colon cancer at age 60 (Figure 2). None of the bladder cases were known smokers.

## Investigation and results

Genetic testing in the form of peripheral blood karyotype and array cytogenetic analysis was normal in each proband. Immunohistochemical analysis for tumour expression of mismatch repair proteins MLH1, MSH2, MSH6 and PMS2 was normal in the proband in each family.

## Discussion

The occurrence of two families with three or more affected cases with UCC is extremely rare. There is no evidence that the two families are related. The most likely mode of inheritance in both families is autosomal dominant, with the father in family A possibly dying before symptoms developed. Autosomal recessive inheritance would also be possible in family A and cannot be excluded. Familial TCC is not widely documented; however the few documented familial cases in three families identified in the older literature show much earlier onset of disease than in our families (Fraumeni and Thomas 1967; McCullough et al. 1975; Ilic et al. 2011). Mueller et al. reviewed cases of UCC and identified two cases of later onset UCC with a similar phenotype to our cases (Mueller et al. 2008), with normal testing for HNPCC genes.

DNA has been stored on key siblings for the future identification of candidate genes. So far, no dominantly inherited genes have been implicated in late onset UCC; the interaction between genetic and environmental factors makes their identification challenging. Polymorphisms in genes involved in metabolism of environmental toxins are known to modify individual susceptibility (McCullough et al. 1975).

All the cases we describe have a later onset UCC bladder phenotype. It is unclear whether the other cancers in family B are related or more likely are sporadic occurrences. Urothelial cancers are estimated to occur in up to 18% of MSH2 gene carriers (van der Post et al. 2010; Skeldon et al. 2013), although these are predominantly upper tract TCC rather than bladder although bladder cancers have been reported rarely and with male predominance. Neither of our families had abnormal mismatch repair proteins on immunohistochemistry. Somatic mutations have been identified in bladder cancers with around 50–60% of cases showing mutations in FGFR3 (Balbás-Martínez et al. 2013). Other genes including STAG2 on the X-chromosome (Solomon et al. 2013) have been recognised, but no germline mutations as yet identified in a pure bladder cancer phenotype.

### Screening

A family history of UCC causes a two-fold increase (Fraumeni and Thomas 1967; McCullough et al. 1975), in the risk of its occurrence among first degree relatives. The risk is less for more distant relatives. The risk is exacerbated by environmental exposures, particularly smoking, and relatives should be made aware of this.

There is no active screening programme at present for such cases. We have advised relatives in these families to watch closely for symptoms of bladder cancer, (e.g. haematuria), and seek urgent medical attention if any symptoms occur. We recommend regular screening cystoscopy every 3 years, from the 5th decade. Haematuria, or other suspicious symptoms, should prompt discussion with a general practitioner and referral for urgent cystoscopy if needed. Careful taking of a family history and testing for mismatch repair protein tumour immunoexpression may help identify or exclude HNPCC as a cause. In both families, the later onset cases had a higher stage of bladder cancer, and the screened siblings were identified at an earlier age and earlier staging. Treatment was not materially different from sporadic cases. Screening of family members may allow earlier detection of bladder cancers at an earlier stage and improve treatment options and success of treatment.

## Conclusion

UCC bladder may occur with familial autosomal dominant inheritance. We describe two additional families to the existing two late onset HNPCC negative families in the literature (Mueller et al. 2008), confirming a autosomal dominant phenotype distinct from early onset UCC. We suspect that a small proportion of urothelial cell carcinomas of the bladder may have a strong family history. Clinicians should take a thorough family history in cases of bladder cancer and should be aware that some familial cases may present with a later age at onset and incomplete penetrance.

Further research in this area may identify candidate genes, and improve the understanding of the pathogenesis of UCC and, ultimately, lead to improvements in screening and treatment if testing for germline UCC bladder cancer mutations becomes possible.

### Consent

Written informed consent was obtained from the patients for the publication of this report and any accompanying images.
